# Investigating the association between depressive disorders and cerebral haemodynamics

**DOI:** 10.1192/bjo.2021.650

**Published:** 2021-06-18

**Authors:** Tamara Chithiramohan, Jvalant Parekh, Lucy Beishon, Golo Kronenberg, Victoria Haunton, Jatinder Minhas, Thompson Robinson, Ronney Panerai

**Affiliations:** 1 University of Leicester, Department of Cardiovascular Sciences; 2 University Hospitals of Leicester

## Abstract

**Aims:**

Alterations in cerebral blood flow (CBF) may contribute to the development of depression, and serve as a novel biomarker. The aim of this review is to summarise and synthesise the available evidence on alterations in cerebral haemodynamics in depressive disorders relative to healthy control populations.

**Method:**

MEDLINE (1946- present), EMBASE (1947– present), Web of Science (1970–present), PsycINFO (1984–present), CINAHL (1976–present) and CENTRAL were searched using a predefined search strategy. Studies which compared the cerebral haemodynamics of adult patients (>18 years old) with depressive disorders against healthy controls (HC), by any imagining modality, were included. Studies with varying severity and chronicity of depressive disorder were included. A meta-analysis was conducted in four groups: 1) CBF (ml/min/100g) 2) Cerebral blood flow velocity (CBFv) (cm/s) 3) Combined CBF and CBFv 4) Ratio of uptake of radiotracer. A random effects model was used and heterogeneity and publication bias were assessed. Data are presented as mean difference (MD) or standardised mean difference (SMD) and 95% confidence interval (95% CI). A narrative synthesis of the remaining studies was performed.

**Result:**

87 studies met the inclusion criteria. CBF (ml/min/100g) was significantly reduced in patients with depression compared to HC (15 studies, 538 patients, 416 HC, MD: −2.24 (95% CI −4.12, −0.36), p = 0.02, I2 = 64%). There were no statistically significant differences between patients and controls in the other three outcomes. CBFv (cm/s): 6 studies, 305 patients, 198 HC, MD: −1.23 (95% CI −6.10, 3.64, p = 0.62, I2 = 65%. Combined CBF and CBFv: 20 studies, 804 patients, 573 HC, SMD: −0.16 (95% CI −0.32, 0.01), p = 0.06 I2 = 51%. Ratio of uptake of radiotracer: 3 studies, 60 patients, 53 HC, MD: −0.11 (95% CI −0.11, 0.11), p = 1.00, I2 = 0%). The narrative synthesis revealed varying results, with many studies identifying a decrease in CBF in depressed patients compared to controls, but other studies identifying an increase, or mixed results. Multiple regions of impairment were identified, including the anterior cingulate cortex and prefrontal cortex.

**Conclusion:**

There was a statistically significant reduction in CBF in depressed patients compared to controls. The narrative synthesis revealed varying results, however specific regions of interest have been identified. Further research is needed to explore the effect of antidepressant medication, utilising different imaging modalities, and at different levels of disease severity.

